# Extracellular Vesicles as Therapeutic Resources in the Clinical Environment

**DOI:** 10.3390/ijms24032344

**Published:** 2023-01-25

**Authors:** Jorge Sanz-Ros, Cristina Mas-Bargues, Nekane Romero-García, Javier Huete-Acevedo, Mar Dromant, Consuelo Borrás

**Affiliations:** 1 Freshage Research Group, Department of Physiology, Faculty of Medicine, University of Valencia, Centro de Investigación Biomédica en Red Fragilidad y Envejecimiento Saludable-Instituto de Salud Carlos III (CIBERFES-ISCIII), INCLIVA, 46010 Valencia, Spain; 2 Department of Cardiology, Hospital Universitari i Politècnic La Fe, 46026 Valencia, Spain; 3 Department of Anesthesiology and Surgical Trauma Intensive Care, Hospital Clinic Universitari de Valencia, University of Valencia, 46010 Valencia, Spain

**Keywords:** extracellular vesicles, therapy, clinical trials

## Abstract

The native role of extracellular vesicles (EVs) in mediating the transfer of biomolecules between cells has raised the possibility to use them as therapeutic vehicles. The development of therapies based on EVs is now expanding rapidly; here we will describe the current knowledge on different key points regarding the use of EVs in a clinical setting. These points are related to cell sources of EVs, isolation, storage, and delivery methods, as well as modifications to the releasing cells for improved production of EVs. Finally, we will depict the application of EVs therapies in clinical trials, considering the impact of the COVID-19 pandemic on the development of these therapies, pointing out that although it is a promising therapy for human diseases, we are still in the initial phase of its application to patients.

## 1. Introduction

In the last three decades, a new mechanism of intercellular communication, based on the release into the extracellular medium of vesicles surrounded by a lipid bilayer by different cells, has begun to be studied in depth. The first mention of these vesicles appeared in 1967 when Peter Wolf defined platelet powder as the sediment obtained by ultracentrifuging platelet-free plasma [1]. Later, apoptotic bodies, which are vesicles released during the process of apoptosis, capable of functioning as signaling particles for cells of the immune system, were described [2]. In recent years, it has become clear that virtually all cells, healthy or not, release different types of vesicles into the extracellular environment that mediate intercellular communication across several tissues. The different types of vesicles have been given various nomenclatures according to their size and releasing cell: microvesicles, exosomes, ectosomes, endosomes, oncosomes, or microparticles [3].

Extracellular vesicles (EVs) serve as vehicles for the transfer of different molecules between cells (proteins, lipids, DNA, RNA, miRNAs, etc.) and are involved in numerous physiological and pathological processes such as the removal of unwanted proteins, antigen presentation, genetic exchange, immune response, angiogenesis, inflammation, tumor metastasis, and dissemination of pathogens or oncogenes [3,4,5].

The most studied types of EVs are exosomes and microvesicles (MVs). Exosomes are smaller, as they present a size between 30 and 200 nm, are formed inside the cell and accumulate in multivesicular bodies (MVBs). Later, these exosomes are released by the fusion of MVBs with the plasma membrane. On the other hand, MVs are larger, up to 1000 nm in size, although smaller MVs can also be found. MVs are formed directly from the plasma membrane, so theoretically the membrane of these EVs has the same composition as that of the cell of origin [3,6,7]. This nomenclature, however, is not completely accurate from an experimental point of view, as most vesicle isolation methods are based on vesicle size, which does not allow complete differentiation between the origin of the vesicles. More recently, the ISEV (International Society of Extracellular Vesicles) has proposed a nomenclature based on vesicle size: small EVs (sEVs) for EVs traditionally referred to as exosomes (diameter up to 200 nm) and large EVs (lEVs) for EVs traditionally referred to as microvesicles (diameter between 200–1000 nm) [6]. This boundary defining small EVs has varied over the last years between 100 and 200 nm.

The clinical use of EVs has been boosted by the encouraging results of studies in preclinical models of different fields, such as cancer, immune diseases, or regenerative medicine, that have shown the potential use of these vesicles as biomarkers, treatments, or drug delivery systems [8]. These results have led to the development of clinical-grade EVs, and clinical trials using EVs have gained momentum in the last 3–4 years, greatly conditioned by the COVID-19 pandemic [9,10,11]. Here, we will focus on the use of EVs from different sources as a therapeutic approach for human diseases, reviewing the current state-of-the-art regarding clinical trials, and discussing the challenges and limitations that this new avenue of research presents.

## 2. Cell Sources of EVs for Clinical Purposes

### 2.1. EVs Obtained from Body Fluids

**Figure 1:**
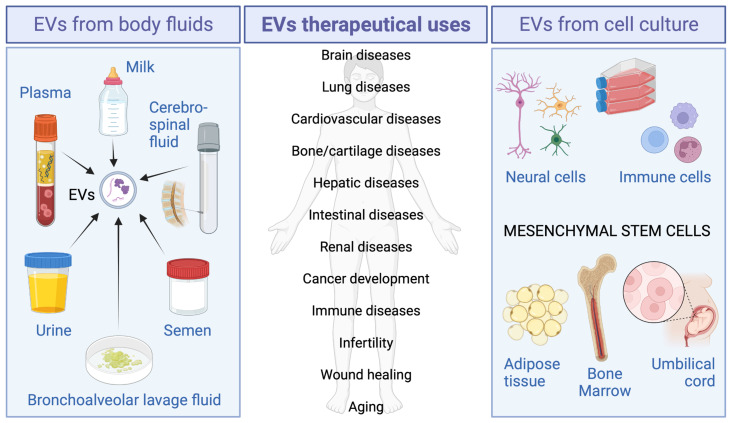
Most used cell sources for EVs obtention and their possible clinical applications.

EVs can be isolated from body fluids such as semen, urine, milk, bronchoalveolar lavage fluid, and plasma, for therapeutic applications (Figure 1). 

Seminal EVs are mainly used for the treatment of infertility as they enhance sperm motility and modulate the implantation process of the embryo [12]. 

Urinal EVs have a major role in the prediction of the response to therapy in urogenital system diseases [13]. Indeed, the identification of specific biomarkers, including protein, lipids, and miRNAs, within urinary EVs unveils the prognosis of the prostate, bladder, and renal cancers [14]. 

Human milk EVs (hMEVs) are safe for oral consumption, which supports their use as a nutraceutical/therapeutic agent [15]. hMEVs are mainly employed in human milk fortification to promote the beneficial effects of maternal breast milk or to improve infant milk formula [16,17]. Moreover, hMEVs have shown a therapeutic role in intestinal inflammation as they enhance tissue repair in a mouse model of preterm infants with Necrotizing Enterocolitis [18]. hMEVs also display bioactive molecules with anti-viral effects, such as dengue [19], and anti-tumor effects through miR-148a-3p, which hampers colon cancer development [20]. 

Airway EVs can be isolated from bronchoalveolar lavage fluid (BALF). BALF-EVs are famous for their inflammatory modulatory properties, which make them valuable tools for the treatment of different lung diseases [21]. As an example, BALF-EVs increase interleukins IL-1β and IL-6, Tumor Necrosis Factor α (TNFα), and CC motif chemokine Ligand 2 (CCL2) in sarcoidosis disease [22,23] and acute respiratory distress syndrome (ARDS) [24]. Further, BALF-EVs improve leukotriene and IL-8 production in asthma patients [25]. 

Human plasma has been used as a therapy since several decades ago [26,27]. Plasma-derived EVs are released from different cells and have been analyzed as therapeutics for several conditions, such as cardiac and bone diseases, wound healing, aging, and muscle atrophy due to their anti-inflammatory, antioxidant, pro-angiogenic, and anti-apoptotic properties [27]. These beneficial effects of plasma-EVs derive from their intrinsic capacity to modulate cell signaling cascades such as Toll-Like Receptor 4 (TLR4), Extracellular signal-Regulated Kinase 1/2 (ERK1/2), and p38 Mitogen-activated protein kinase (P38MAPK) [28], to increase the expression of collagen type III, and to decrease the expression of TGF-β and IL-6 [29]. In addition to regular plasma, modified versions of the regular plasma have also been investigated as a source for therapeutic EVs. Among them, plasma containing additional blood components (platelets), plasma from donors who have undergone specific procedures (ischemic pre-conditioning or exercise training), and plasma from donors with specific characteristics (young age or infection survivors) have been studied [27].

### 2.2. EVs Obtained from in vitro Cell Cultures

EVs can also be isolated from cell cultures for therapeutical purposes. The majority of EVs obtained in vitro come from mesenchymal stem cell (MSC) cultures, and among them, the most widely used are bone marrow MSC, umbilical cord MSC, and adipose-derived MSC (Figure 1).

Human bone marrow MSC-derived EVs (hBM-MSC-EVs) are mostly involved in the treatment of diseases of connective tissues such as cartilage and tendon defects, or osteoarthritis [30]. hBM-MSC-EVs can promote cartilage regeneration through the stimulation of chondrocytes to produce proteoglycans and type II collagen [31]. hBM-MSC-EVs enhance tendon healing by stimulating tendon resident stem cells [32]. In addition, EVs derived from BM-MSCs contain miR-146a, which is a famous anti-inflammatory microRNA. miR-146a suppresses the transcriptional activity of the Nuclear Factor Kappa-light-chain-enhancer of activated B cells (NF-κB) pathway, reducing the expression of the downstream inflammatory factors IL-1β, IL-6, and TNF-α [33].

The therapeutic efficacy of human umbilical cord MSC-derived EVs (hUC-MSC-EVs) has been investigated in many diseases. hUC-MSC-EVs promote nerve regeneration and motor function in the injured areas [34]. Indeed, it has been proved that hUC-MSC-EVs play a neuroprotective role in perinatal brain damage [35,36]. hUC-MSC-EVs are also useful for cartilage injury since they can significantly improve cartilage repair through miRNA-181c-5p [37]. In hepatic pathologies, hUC-MSC-EVs are capable to reduce hepatic inflammation, collagen deposition, oxidative stress, and apoptosis in liver ischemia/reperfusion injury [38,39]. Moreover, hUC-MSC-EVs have shown positive effects on renal disease as they improve kidney function in grade III-IV patients [40]. 

EVs from human adipose tissue-derived mesenchymal stem cells (hAD-MSC-EVs) are effective in a wide range of diseases [41], mainly due to their beneficial properties for tissue repair, reduction in injuries, and antiinflammation. Additionally, this source has advantages in its high availability. Therefore, EVs from this source have a broad application prospect. In a study of osteoarthritis, hAD-MSC-EVs reduced the production of IL-6 and PGE2 [42]. Similarly, a study of allergic asthma showed a reduction in IL-5 levels in lung tissue treated with hAD-MSC-EVs [43]. In a study of atopic dermatitis, hAD-MSC-EVs reduced mRNA expression of various inflammatory cytokines such as IL-4, IL-23, IL-31, and TNF-α [44]. In the aspect of wound healing, hAD-MSC-EVs were able to increase the gene expression of *N-cadherin*, *cyclin-1*, *Proliferating Cell Nuclear Antigen (PCNA)*, and *collagen I, III* [45,46]. 

Although performed using mouse AD-MSC-EVs, the following studies display the versatility of these EVs. In one study, AD-MSC-EVs administration proved to modulate the apoptotic function of mutant Huntingtin aggregates, thereby hampering Huntington’s disease (HD) progression [47]. In an Alzheimer’s disease (AD) mouse model, it was also revealed that AD-MSC-EVs reduced b-amyloid deposits and neuronal death [48]. Finally, we have recently shown the beneficial effect of young mice-derived AD-MSC-EVs for antiaging purposes as they recovered muscle and kidney functions [49].

On the other hand, EVs can also be isolated in vitro from neural cells (such as neurons, oligodendrocytes, microglia, and astrocytes) and immune cells (macrophages, dendritic cells, T cells, and natural killer cells) for therapy (Figure 1).

Neural cells including neurons, astrocytes, oligodendrocytes, and microglia release EVs for neural communication but are also involved in the propagation of various central nervous system (CNS) diseases [50,51,52]. However, cerebrospinal fluid (CSF) collection for selective isolation of neural EVs is quite challenging because of the invasive nature of the procedure and the small size of the obtained samples [53]. For instance, studies performed in animals have revealed that astrocytic EVs are enriched in miR-873a-5p, which inhibits ERK phosphorylation and the NF-κB signaling pathway, thus promoting the release of anti-inflammatory factors from microglia [54]. Similarly, microglial EVs-derived miR-124 promotes the activation of microglia in traumatic brain injured tissue during the acute stage, thus reducing the neuroinflammatory response and restoring neural function [55].

Depending on the parental cell type and activation status, immune cell-derived EVs can induce either immune-stimulatory or immune-suppressing responses and therefore can play dual roles in physiological and pathological processes. Regarding their immune-activating properties, both CD8+ T cell-derived EVs and dendritic cell-derived EVs are being used as antigen delivery tools to hamper tumor growth in metastatic melanoma and non-small cell lung cancer patients [56,57,58]. On the other hand, the immunosuppressive features of immune cell-derived EVs make them also suitable as therapeutic agents in transplantation tolerance and autoimmune diseases. As an example, immature dendritic cells-derived EVs reduce the expression of Major Histocompatibility Complex (MHC) molecules, which helps to prevent graft rejection in models of cardiac and intestinal transplantations [59,60,61]. Similarly, regulatory T lymphocyte-derived EVs have immunomodulatory effects that improve transplantation tolerance [62].

### 2.3. Body Fluids Versus Cell Cultures as Sources of EVs

The therapeutical potential of EVs is highly related to their uptake by target cells to achieve the desired outcomes. EVs obtained from different cell sources and donors may have different biological cargos, thus exerting different responses on recipient cells [63]. It has been demonstrated that EVs’ internal cargo has a similar pattern to the donor cell, suggesting a selective packaging of the cellular components into EVs [64]. More specially, superficial cargos play a role in EVs uptake. Indeed, specific molecules on the EVs surface might drive the cell-specific receptors for targeted EVs uptake [65]. Hence, the selection of an appropriate cell source could improve EVs targeted uptake. 

The isolation of EVs directly from body fluids compared to cell culture media is more time-efficient, cost-efficient, and has relatively higher yields [66]. The choice of the EV isolation method significantly affects EV yield from blood samples, together with lipoprotein and protein contaminants [67]. Moreover, it has been reported that an increased yield is coupled with a short processing time of the sample [68]. Further, obtaining EVs from body fluids eliminates the need for exogenous cell culture media reagents that are not suitable for clinical-grade manufacturing. Indeed, cell culture media are prone to contamination and thus, contaminant removal must be addressed with an extra purification step [69]. On the opposite, in vitro cell culture facilitates EVs engineering to use them as drug delivery carriers. Lastly, the isolation of EVs from body fluids has some disadvantages, including a more heterogenous population and containing high levels of contaminants [27].

## 3. Modifications for Enhanced EVs Production and EVs’ Cargo Modulation

EVs large-scale production is a critical point for the translation from the bench to the bedside application of EV-based therapies. The “massivEVs” workshop announced the minimum requirements that must be reached towards EVs production in compliance with good manufacturing practices (GMPs), product validation, and regulatory issues [70].

For instance, using a 3D bioreactor was generally accepted as the most efficient method for the large-scale production of EVs from MSCs [71]. 

Different conditions can also be applied to promote EVs production [72]. For example, stimulation by shear stress [73], high-temperature [74], low oxygen tension [75], or ethanol [76] might increase EV production and shedding. However, the function of these EVs on recipient cells might be different. It has been reported that MSC culture in low oxygen conditions (1–5% O_2_) increases the number of EVs released and their cargo composition (growth factors, antioxidant enzymes, and miRNAs), thus enhancing their pro-angiogenic, immunomodulatory, anti-senescence, and antioxidant effects [77,78,79].

On top of that, therapeutic agents can be incorporated into EVs through the exogenous loading of drugs [80,81].

Exogenous loading of naive EVs isolated from body fluids or cultured cell media can be conducted by a variety of methods. Among these procedures, the most used are as follows:

- Co-incubation with a drug at different temperatures [82,83]: EVs are incubated with a saturated solution of the desired drug which will diffuse inside EVs to equalize the concentrations. This method is relatively simple to scale up and enables to work of highly fragile therapeutic molecules, although it has a low loading efficiency.
- Sonication of EVs with a drug mixture [84,85,86]: Treatment with ultrasounds produces transient pores in EV membranes that allow drug diffusion inside the vesicles. This method has a higher loading efficiency but might result in the destruction and inactivation of some therapeutic molecules.
- Electroporation of EVs [87,88]: This technique is used to introduce siRNA and miRNA within EVs, but also anticancer drugs such as Paclitaxel, or antibiotics such as Doxycycline. Electroporation may result in the aggregation of RNA particles that will not be incorporated into EVs [89]. Therefore, the last step to ensure an efficient purification of loaded EVs from free RNA aggregates should be performed to obtain reliable and reproducible therapeutic effects.
- Transient permeabilization of EVs membranes with saponin [90]: EVs are incubated with saponin to selectively remove membrane-bound cholesterol, creating transient pores in the EV membrane, thus promoting drug loading. This method is used for enzymes and other protein molecules.

Engineered EVs can be mass-produced by the fusion of the surface of EVs with lipid-based materials (DOTAP, POPC, DPPC, and POPG) using an extrusion technique to form a hybrid lipid membrane structure. Interestingly, this modification increased the number of released EVs from 6- to 43-fold, and their uptake by target cells (lung cancer cells A549) was 14-fold higher compared to non-engineered EVs [91]. Similarly, a recent study reported that both small and large EVs have very low uptake and fusion with target cells (fusogenic activity) that can be ameliorated using a fusogenic protein such as the glycoprotein G of the vesicular stomatitis virus (VSV-G) [92].

On the other hand, exogenous loading of parent cells is mainly performed by two methods: the loading of small therapeutic cargo into cultured parent cells, and the transfection of parent cells with therapeutics encoding DNA plasmids.

- The first method consists of incubating the parent cells with small therapeutic agents, which will be incorporated by the cells and then included in the cargo of the released EVs. This method has been used for loading pancreatic adenocarcinoma cells in vitro with curcumin, which has known anticancer and anti-inflammatory properties [93]. The pros of this approach are that it allows the manufacturing of large batches of EV-based formulations. The cons include high amounts of the drug required for the loading of the parent cells, which is partially metabolized by the cells, thus, lowering the amount of drug loaded onto EVs, which may lead to reduced therapeutical effects.
- The second method is the transfection of parent cells with plasmidic DNA encoding the desired therapeutic molecule, followed by the isolation of loaded EVs from the media. This method has been reported to load macrophages with plasmidic DNA encoding catalase. Loaded EVs collected from cell media displayed encapsulated plasmidic DNA, mRNA, and the encoded therapeutic active catalase [94].

In general, the production of exogenous drug loaded-EV formulations through parental cell modifications seems to be more reproducible than endogenous loading of naive EVs.

## 4. Methods to Prepare Clinical-Grade EVs

When facing EVs as a therapeutic agent, several key aspects must be addressed, such as their isolation methods, storage conditions, and administration route toward their clinical translation.

### 4.1. Isolation Methods

Many techniques have been developed for EVs isolation and have been extensively reviewed elsewhere [95,96,97,98]. These techniques are mostly based on EVs biophysical and/or biochemical characteristics, such as size, density, shape, or specific surface markers. It has been described that each isolation technique has the potential to affect the structural integrity and functional activity of EVs [99]. Therefore, the election of the most appropriate method to isolate EVs is of utmost importance for a deep understanding of their biological function before their use in clinics. 

Isolation techniques are constantly being improved and combined to provide a strategy with high purity, high yield, and structural and functional integrity. Currently, the most frequently used EVs isolation techniques are briefly described below:

- Ultracentrifugation (UC): Differential ultracentrifugation, with or without density gradients, is the most used and was traditionally considered the “gold standard” technique for EVs isolation [99,100], which combines different densities and gradients with different centrifugal speeds and forces. Differential ultracentrifugation has some disadvantages, such as being not scalable, time-consuming, and resulting in impure EV preparations, which leads to EV aggregation. However, sequential ultracentrifugation has the great advantage that it can be used for large-scale production of clinical-grade MSC-EVs [101].
- Size Exclusion Chromatography (SEC): This method uses a chromatographic column with a porous stationary phase made of polymers. EVs are separated according to their size, which defines the time to elute from the column [102]. SEC has several advantages; among them, the highest purity is GMP-compliant and is a scalable system [103].
- Precipitation: Hydrophilic polymers, such as polyethylene glycol (PEG), are usually used as highly hydrophilic polymers that interact with surroundings to create a hydrophobic microenvironment, thus enabling the precipitation of the EVs [100,102,104,105]. Although the precipitation method has a lower purity and higher contamination, it has many advantages: low price, rapid EV extraction, and high yield, especially for large-scale applications.
- Ultrafiltration (UF): UF uses membranes with molecular weight cut-offs ranging from 10–100 kDa, which enables the separation of EVs according to their size. Ultrafiltration has a great advantage as it reduces isolation times and costs compared with other techniques [106,107]. However, UF has lower yields and purities due to the interaction between vesicles and the filtration membranes that creates aggregates that ultimately block the pores.
- Tangential Flow Filtration (TFF): TFF couples permeable membrane filtration and flow to obtain EVs from a colloid matrix. TFF enables the concentration of EVs from large volumes of samples in short periods (1 hour). Compared to UC, TFF has a higher yield, fewer aggregates, and better batch-to-batch consistency [108].
- Immunoaffinity capture: This technology is based on EVs membrane surface protein markers such as CD9, CD63, CD81, CD82, and other cell adhesion molecules [109]. Thus, this technique enables the separation of specific EVs subtypes. The main limitation of this method is that it can only detect surface markers, and the antibodies are expensive.
- Microfluidics: Microfluidic devices combine EVs isolation (size and affinity-based) and detection in miniaturized chips with the benefits of reduced time, high sensitivity, specificity, and high production [110,111]. They have some disadvantages such as highly complicated devices, expensive prices, and the need for specialized equipment.

It has been suggested that homogeneous populations of EVs would be safer [112]. Therefore, the isolation of a monodisperse EV population with a smaller size may improve EV uptake by recipient cells and its subsequent therapeutic effects. In addition, EV preparations may include different co-isolates (such as aggregates and precipitates) and exogenous contaminants related to the starting sample or the manufacturing process. Indeed, it has been highlighted that EV purity may not correlate with EV functionality because the co-isolates can contribute to the stability and biological activity of EVs [113,114]. Further, the presence of contaminants could affect EV uptake. Therefore, highly purified EVs appear to have preferential uptake by cells [115]. 

Concerning clinical applications of EVs, the major challenge is to isolate EVs with high yield and high purity, while preserving their intact structure and biological activity. Preferably, the isolation method should be scalable, cost-effective, and with a high-throughput production process [97]. As mentioned above, all the currently used isolation techniques differ in yield and purity [116]. These methods should be improved to obtain highly purified EVs for preferential uptake in the therapeutic context. In this regard, a combination of several extraction and purification methods should be considered.

### 4.2. Storage Methods

The storage of EVs is critical to preserve their stability and biological activity. There is still no consent regarding the best method to store EVs, although it is generally accepted that temperature, fluid characteristics, and freeze–thawing cycles deeply influence their conservation [117].

Some studies have reported that it is not practical to directly isolate EVs from a patient sample immediately after biofluid collection. Therefore, the biofluid storage method has also been addressed. Seminal plasma can be stored at -80 °C for short periods without significantly impacting EV yield or bioactivity [118,119]. Moreover, seminal EVs morphology, concentration, and size after short (2 years) and long-term freezing period (30 years) of semen at −80 °C was not affected [120]. Urine stored at −80 °C guaranteed effective preservation of urinal EVs, whereas storage at −20 °C resulted in a significant loss of EVs compared to fresh urine [121]. The storage of plasma at 4 °C, −20 °C, or −80 °C did not result in significant degradation of EV cargo [122]. Interestingly, it has been reported that storage of plasma at room temperature for over 42 h or at −80 °C for 12 years was not accompanied by a degradation of EVs RNA content [123]. BALF storage conditions can also destabilize the morphological features of EVs. The protein content is altered due to the dissociation of membrane-integrated proteins, rather than the loss of internal EV proteins [124].

Regarding EVs produce from in vitro cell cultures, culture media can be stored without altering EV concentration, which remained stable after one week of storage at 4 °C, −20 °C, and −80 °C. However, EV miRNA levels decreased during this period, especially when the media were stored at 4 °C and −20 °C. After 30 days of storage, the EV miRNA content dropped to below 50% compared to the initial amount [125,126].

On the other hand, other studies have addressed the impact of different storage conditions on isolated EVs. In general terms, storage at −80 °C is encouraged. It is accepted that storage at 4 °C and −20 °C can affect EV size and number, and cause EV aggregation [127]. The possibility to freeze-dry isolated EVs for long-term storage at room temperature has been addressed. Freeze-drying might help to preserve EV characteristics and function, and thus might offer a cost-effective storage strategy because it reduces transport costs [128]. In addition, EVs resuspension buffer is critical. Phosphate-buffered saline is the most used for EV resuspension. However, EV aggregation is an inherent consequence of storage at −80 °C which might alter their structure and biological function [39]. A colloidal solution with polymers could prevent the aggregation of EVs [40] and lead to the preservation of biological activity after their uptake [96]. Other possibilities include the addition of disaccharide stabilizers to the buffer to improve EV preservation, such as trehalose [129], mannitol [130], or dimethyl sulfoxide (DMSO) [126]. All three have proven to maintain EV integrity and function.

Taken together, for clinical applications, EVs should be suspended in sterile 0.9% NaCl and stored at −80 °C. EV products should be formulated for single-use because it has been observed that their number decreases and their morphology and content are altered after two cycles of freezing and thawing, which in turn affects their uptake by recipient cells [131]. Finally, independently of the storage formulation and conditions, batch stability will have to be carefully examined and monitored during storage [132]. 

### 4.3. Delivery Methods

The selection of the EV administration route is determined by the type of disease. For example, intra-articular injection is recommended for the treatment of osteoarthritic joints and periocular injection is preferred for retinal diseases. Nevertheless, EVs have the intrinsic capacity to cross the blood-brain barrier and can therefore be administered upon intravenous (IV), intraperitoneal (IP), subcutaneous (SC), or intranasal (IN) delivery to treat a broad range of CNS pathologies [133]. Many studies have reported that EVs can be successfully administered both systemically and locally and exert their effect at the desired place. 

Systemically administered EVs have proven to successfully reach metastatic cells all over the organism [134], and their uptake was positively correlated with their dosage [135]. However, this route of administration has the inconvenience that EVs can also be uptaken by macrophages in the reticuloendothelial system, thereby promoting EVs clearance [136,137]. Therefore, EVs clearance by macrophages should be decreased to maximize their uptake by target cells and to ensure a high therapeutic effect. Systemic administration of EVs includes intravenous (IV) injection, subcutaneous (SC) injection, intranasal (IN) administration, and oral administration.

SC injection of EVs is the preferred route for wound healing applications. Indeed, SC injection of EVs effectively restored epidermal barrier function [138] and accelerated skin wound repair [139] in animal models. 

IN administration of EVs has been used in animal models to treat epilepsy [140], Parkinson’s disease [141], and Alzheimer’s disease [142], among other neurological disorders. Recently, aerosol inhalation administration of EVs has been documented in various clinical trials as a treatment for COVID−19 disease [143,144,145] and other respiratory pathologies [146]. 

Milk-derived EVs, plant-derived EVS, and bacterial-derived EVs have the potential to cross the gastrointestinal tract barrier, which makes them promising delivery vehicles for orally administered drugs [147]. For instance, milk-EVs can remain unaltered in the gastric acidic environment thanks to their unique lipid composition and membrane rigidity [148,149]. In addition, this lipid bilayer of EVs protects the cargo from enzymatic degradation [150,151]. EVs are mainly absorbed through a transcellular pathway, but they can also be internalized through phagocytosis, pinocytosis, and endocytosis [152,153]. Once absorbed, they are transported by blood and/or lymph throughout the whole organism. Interestingly, orally delivered EVs also influence the immune cells in the digestive tract, which in turn induces local effects [154]. For instance, milk-EVs effects have only been tested in animal models [155].

Compared to systemic administration, local administration provides higher concentrations of EVs to the site of injury and increases EVs uptake by target cells, such as in the knee joint space. However, even faster clearance of EVs was observed and, therefore, repeated administration was needed to ensure the therapeutic effect in a rat model of osteochondral defects [156].

Taken together, it is of utmost importance to select the best administration route for each specific disease to ensure a successful treatment. Regarding clinical application, efficiency, ease of use, safety, and cost must be taken into consideration when choosing the route of administration.

## 5. Current Use of EVs in Clinical Trials and Human Diseases

For this review, we have analyzed all the clinical trials registered in clinicaltrials.gov in which the primary intervention is the treatment of a concrete disease or condition with EVs. In all described trials, EVs come from a specific cell type, tissue, or biological fluid, whether these cells or tissues have been modified or are being used as drug carriers. Thus, we have excluded trials that include the use of synthetic particles or liposomes that carry a specific drug or component. As this review focuses on the therapeutic properties of EVs, we have also excluded trials dedicated to the basic analysis of EVs or that use EVs as biomarkers [157].

Using a search that includes the words “extracellular vesicles”, “exosomes”, “microvesicles”, “ectosomes” and “microparticles”, we found a total of 383 registered clinical trials. We then identified 60 unique clinical trials that use therapy with EVs as the primary intervention (Table 1). Therefore, although the use of EVs as a therapeutic tool is rising, the number of studies that analyze EVs, use EVs as biomarkers or have them as a secondary measurement is much higher.

The COVID-19 pandemic has shaped the way EVs therapy has developed in clinical trials, as 51 of these 60 trials have been registered in the last 4 years. This has biased the use of EVs as a treatment of COVID-19-related syndromes or lung pathologies, in part due to the absence of standard treatment for COVID-19 at the beginning of the pandemic. Easier recruitment of patients with lung pathologies, as well as hampered recruitment for other diseases, have made COVID-19 the predominant disease to treat in EVs human trials (Table 1). This is likely to change in the future, as the COVID-19 pandemic resolves and the application of EVs broadens to other conditions and organs.

### 5.1. Trial Status

**Figure 2:**
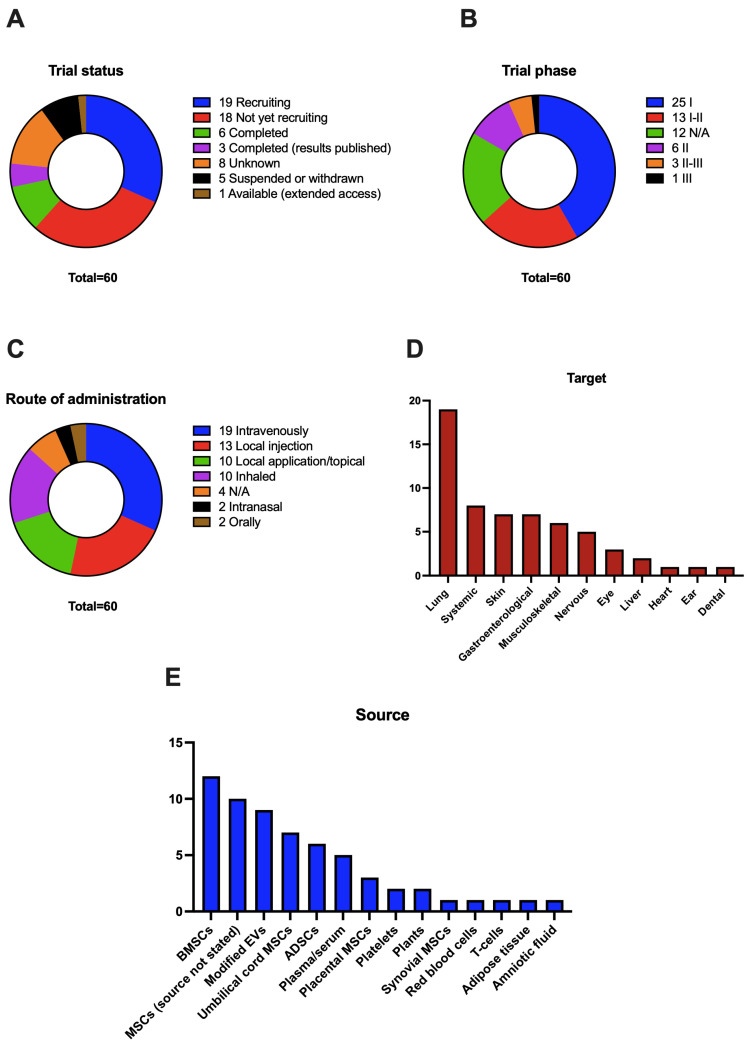
Graphical representation of the analysis of Table 1 results regarding ongoing and finalized clinical trials that use EVs therapy as the main intervention. (**A**): Trial status defined by clinicaltrials.gov. (**B**): Trial phase defined by clinicaltrials.gov, when the phase is not available or is not applicable N/A is shown. (**C**): Route of administration of the EV preparation, when not stated N/A is shown. (**D**): Organs or systems related to the primary outcome measured in the trial. (**E**): Source of the EVs used in the trial.

Regarding trial status (Figure 2A), only nine trials have been completed; of these, three of them have had their results published in peer-reviewed journals. Two of the published results are safety studies of several doses of nebulized EVs. In the first one, the authors demonstrate the efficacy of inhaled EVs from human ADSCs in a preclinical model of lung injury and test the safety of one dose of 2 × 10^8^ to 16 × 10^8^ particles of EVs from hADSCs in healthy adults, which was well-tolerated in all volunteers [146]. In the second study, authors studied the safety of daily doses of 2 × 10^8^ inhaled particles of EVs from hADSCs for 5 days in seven COVID-19 patients, with no evidence of adverse events [144]. The third published study is a randomized controlled clinical trial with 11 patients per group, that shows some efficacy of autologous platelet and EV-enriched plasma in the treatment of chronic temporal bone inflammation after surgery when compared to standard treatment [158]. Other completed clinical trials, with unpublished results, include two more studies on COVID-19, an ex vivo study of blood coagulation, a trial on the safety of intra-discal injections of plasma enriched with platelets and EVs for low back pain, an oral treatment with curcumin-loaded EVs from the ginger plant on inflammatory bowel disease, and a safety study of a topic ointment that includes EVs from MSCs. On the other hand, most of the studies are still recruiting patients or have not started the recruitment, and five of them have been suspended, mainly due to recruitment problems or lack of funding; it is important to highlight that none of the studies have found safety-related issues.

### 5.2. Trial Phase

When analyzing the trial phase (Figure 2B), it becomes evident that we are still in the initial stage of EVs therapeutics, as 60% of the studies are phase I or I-IIa and include a small number of individuals, mostly related to the safety of the treatments, and just some of them use a small comparator group. There is only one study in phase III (NCT05354141), approved in April 2022, that will involve 400 patients with COVID-19-related ARDS; patients will be treated intravenously with 1,2 × 10^9^ EVs from bone marrow MSCs. This study is based on two earlier trials that have been carried out by the same company. Three other trials have some characteristics of a phase III study, that will focus on the treatment of moderate COVID-19 (NCT05216562), retinitis pigmentosa (NCT05413148), and chronic middle ear infections (NCT04761562).

### 5.3. Route of Administration

In terms of the route of administration (Figure 2C), intravenously is the preferred one, with 30% of the studies using it. This approach has some advantages, it is widely used in preclinical models of diseases, and, as we are yet to determine the mechanism of action and delivery to target organs of EVs, IV injection secures a systemic distribution of EVs [159]. Still, the use of IV injections in humans is tightly regulated compared to other topical routes. In the second place comes the use of local injections in the desired target, such as intra-articular or epineural injections. The main advantage of this proposal is that it secures the arrival of EVs to the intended site and minimizes the secondary effects on other tissues. However, some of the organs or tissues, such as the brain, are difficult to reach, and these injections may in part miss the beneficial systemic effects of EVs. Local or topical application to an accessible tissue such as the skin is another interesting approach, mainly for skin regeneration and immune-mediated diseases, which can maximize the effect on the skin while reducing systemic absorption. We still need more studies on the degree of penetration of EVs in wounds and intact skin and how this affects its therapeutic effects [160,161]. The COVID-19 pandemic has increased the number of studies that use the inhaled route for EVs administration. This route seems to be ideal for lung diseases, as EVs can be included in aerosol liquid and delivered to the bronchi and alveolus, to exert their therapeutic effect [162,163]. It is of interest to note the oral administration of EVs; there are only two trials that use this route, but this may be an appealing approach to treat gastroenterological diseases, such as inflammatory bowel disease [164]. Indeed, some studies have highlighted the potential of oral-administered extracellular vesicles to treat systemic diseases, as these vesicles can reach the circulation and distant organs such as the liver or spleen [154,158].

### 5.4. Targeted Tissues and Diseases

Many different diseases are being studied in clinical trials using EVs therapeutics; we have divided trials into the organs or tissues intended to treat (Figure 2D). Currently, lung diseases are the most common in trials, as we have stated before. The COVID-19 pandemic has boosted trials in this field, being the most studied disease. Other lung diseases studied include ARDS and non-COVID-19 pulmonary infections. We have included systemic affections as the second most studied ones, referring to conditions such as solid organ transplantation, metastatic cancer, familial hypercholesterolemia, or intensively ill children. Skin is the third most studied tissue in these trials. The main application is for wound healing and regeneration, appearing from different origins, such as burn wounds or venous trophic lesions, and immune-mediated diseases such as psoriasis. Along with the skin, gastroenterological diseases are the third most studied condition, with trials mainly focusing on inflammatory bowel disease, including Crohn’s and ulcerative colitis, and its complications, mainly perianal fistula. The clinical research on musculoskeletal conditions includes osteoarthritis, bone defects, or meniscal injury. Regarding the nervous system, clinical trials include diverse conditions, such as Alzheimer’s, depression, neuralgia, or stroke. To conclude, other least studied tissues and organs include disorders ranging from macular holes and retinitis pigmentosa in the eye to myocardial infarction or middle ear infections.

### 5.5. Source of EVs

The source of choice to obtain the EVs for most of the studies are MSCs from different tissues, mainly bone marrow, umbilical cord, and adipose tissue (Figure 2E). The experience in the clinical research environment with these cells—leading to the development of clinical grade, ready-to-use cells—along with their regenerative and immunomodulatory properties, with a lower chance of immune rejection, has shifted the use of whole cells to concrete factors released by these cells such as EVs. EVs from MSCs have shown great potential in preclinical trials, mainly improving the regenerative potential of the tissues, and serving as anti-inflammatory and immunomodulatory factors. Plasma or serum, and other biological fluids, are also being used in clinical trials as a source of EVs, however, the use of these fluids makes it more challenging to obtain a pure preparation of EVs. Of particular interest is the use of modified EVs and whether these modifications affect the cells of origin or the vesicles themselves; these modifications aim to improve the therapeutic power of the EV preparation. The most used modification is loading the vesicles with some drugs or factors; in the first registered study, researchers tried to use EVs from erythrocytes loaded with methotrexate to treat malignant ascites (NCT03230708). Another study with preclinical evidence [101,165] is using EVs from BMSCs loaded with a siRNA directed to G12D mutated KRAS to treat metastatic pancreatic cancer (NCT0308631). EVs from MSCs loaded with miR-124 are being studied for the treatment of ischemic stroke [166] (NCT03384433). Three trials using EVs from cells that overexpress the CD24 receptor to treat COVID-19-related conditions (NCT04747574, NCT04902183, NCT04969172). Researchers will use EVs from BMSCs loaded with LDL receptor mRNA to treat familial hypercholesterolemia [167] (NCT05043181). Ultimately, a recent study will study the effect of EVs from HEK-293 cells loaded with an antisense oligonucleotide directed to STAT6 in advanced hepatocellular carcinoma and metastasis from gastric and colorectal cancer [168] (NCT05375604).

## 6. Conclusions

The field of EVs has experienced an unprecedented rise in the last decade [169]. Originally seen as merely cellular waste products, these information carriers are being exhaustively studied in cell biology and intercellular communication [170]. We are still learning the basic cellular processes that give place to EVs [7], however, their application in many diseases is rapidly growing—even faster than our knowledge of the basic biology of EVs. Their particular mechanism of action, carrying molecules from the original cells that can be detected in systemic fluids such as plasma, has led to the study of EVs as diagnostic tools and biomarkers for many diseases, including the development of liquid biopsies based on EVs analysis [171]. 

Due to their innate ability to reach different tissues and cells, these vesicles are excellent drug carriers that can deliver these drugs, for example, to cancerous cells [172]. However, EVs can have a therapeutic effect themselves, especially those coming from the stem or progenitor cells [48]. This has led to the development of multiple vesicle-based therapies targeting diseases affecting humans. Results in preclinical models of these diseases have been generally encouraging, especially in fields such as regenerative medicine and immune diseases, as these vesicles appear to have pro-regenerative and immunomodulatory properties [173], as well as in cancer [174], given the vesicles’ tropism for cancer tissues [175]. There are currently multiple companies developing EVs for clinical use, and there are already several clinical trials testing the safety and efficacy of EVs in various conditions (Table 1 and Figure 2). 

Nonetheless, it must be said that we are still at a very early stage, as most of the studies are in early phases, and there is not a single completed phase III study. For the moment, the disease that is the target of the largest number of trials is COVID-19, partly due to the ease of testing new therapies for this disease, and the greater funding it has received in recent years. It is to be expected that in the coming years, the number of studies in this regard will decrease, increasing the number of studies related to other diseases. 

As for the preferred route of administration, at the moment it is intravenously, closely followed by the local administration at the site of interest, either by injection or topical treatment. Much research remains to be done on the optimal methods for isolating, storing, and delivery of the vesicles, as well as the preferred dose. Due to the effort needed for isolating EVs, few studies perform a comprehensive dose-response study, and even fewer in humans, so these studies need to be developed to find out the optimal methods and doses to obtain clinical-grade EVs and achieve success in clinical trials to make EVs the medicine of tomorrow.

## Figures and Tables

**Table 1:** Registered clinical trials (clinicaltrials.gov) related to therapy with extracellular vesicles (extracellular vesicles/exosomes/microvesicles search) as the main intervention. ADSCs: Adipose-derived stem cells; ARDS: acute respiratory distress syndrome; ASO: anti-sense oligonucleotide; BMSCs: Bone marrow-derived stem cells.

Clinical Trial Identifier	First Posted Date	Disease or Condition	Product	Delivery	Comparators	Phase	Status
NCT05523011	31/08/2022	Healthy adults	Ointment with EVs from MSCs	Topical	Single group	I	Completed
NCT05387278	24/05/2022	Moderate-to-severe COVID-19-associated ARDS	EVs from the placenta and umbilical cord-derived MSCs	Intravenously	Placebo vs. EVs	I	Recruiting
NCT05375604	16/05/2022	Advanced hepatocellular carcinoma and liver metastasis from gastric and colorectal cancer	EVs from HEK-293 loaded with STAT6 ASO	Intravenously	Single group	I	Recruiting
NCT05215288	31/01/2022	Abdominal solid organ transplant with risk of worsening allograft function	EVs from BMSCs (ExoFlo)	Intravenously	Expanded access protocol	Early phase I	Not yet recruiting
NCT05176366	04/01/2022	Refractory ulcerative colitis	EVs from BMSCs (ExoFlo)	Intravenously	10 doses of EVs vs. 15 doses of EVs	I	Not yet recruiting
NCT05130983	23/11/2021	Refractory Crohn’s disease	EVs from BMSCs (ExoFlo)	Intravenously	10 doses of EVs vs. 15 doses of EVs	I	Not yet recruiting
NCT05078385	14/10/2021	Burn wounds	EVs from BMSCs (AGLE-102)	Topical	Single group	I	Not yet recruiting
NCT05060107	28/09/2021	Knee osteoarthritis	EVs from umbilical cord-derived MSCs (CelliStem)	Intraarticular	Single group	I	Not yet recruiting
NCT05043181	14/09/2021	Familial hypercholesterolemia	EVs from BMSCs enriched with LDL receptor mRNA	Abdominal puncture	Single group	I	Not yet recruiting
NCT04849429	19/04/2021	Chronic low back pain	Platelet-rich plasma enriched with EVs	Intra-discal injection	Placebo vs. EVs	I	Completed
NCT04747574	10/02/2021	Moderate-to-severe COVID-19	EVs over-expressing CD24 from T-REx-293 cells	Inhaled	Single group	I	Recruiting
NCT04664738	11/12/2020	Skin graft donor site wound	EVs from platelets (PEP)	Topical	Standard treatment vs. several doses of PEP and TISSEEL	I	Enrolling by invitation
NCT044491240	29/07/2020	COVID-19 pneumonia	EVs from MSCs	Inhaled	3 doses of EVs	I	Completed
NCT04389385	15/05/2020	Early-stage COVID-19 pneumonia	EVs from COVID-19 specific T cell	Inhaled	Single group	I	Unknown
NCT04388982	15/05/2020	Alzheimer’s disease	EVs from ADSCs	Intranasal	3 doses of EVs	I	Unknown
NCT04327635	31/03/2020	Acute myocardial infarction	EVs from platelets (PEP)	Intracoronary	3 doses of intracoronary EVs	I	Recruiting
NCT04313647	18/03/2020	Healthy adults	EVs from ADSCs	Aerosol inhalation	Several doses of EVs	I	Completed, results published in [146]
NCT04276987	19/02/2020	COVID-19 pneumonia	EVs from ADSCs	Inhaled	Single group	I	Completed, results published in [144]
NCT04270006	17/02/2020	Periodontitis	EVs from ADSCs	Local injection	Single group	Early phase I	Unknown
NCT04202783	18/12/2019	Craniofacial neuralgia	EVs from neonatal stem cells	Epineural injection and intravenously	Single group	I	Suspended due to the COVID-19 pandemic
NCT03857841	28/02/2019	Preterm neonates at high risk for bronchopulmonary dysplasia	EVs from BMSCs (UNEX-42)	Intravenously	Placebo vs. 3 different doses of EVs	I	Terminated due to business decision
NCT03608631	01/08/2018	Metastatic pancreatic cancer with KRAS G12D mutation	EVs from BMSCs loaded with KRAS G12D siRNA	Intravenously	Single group	I	Recruiting
NCT03437759	19/02/2018	Macular holes	EVs from umbilical cord-derived MSCs	Intravitreal	Standard treatment vs. standard treatment + EVs	I	Active, not recruiting
NCT02565264	01/10/2015	Wound healing	EVs from plasma	Topical	Single group	Early phase I	Unknown
NCT01294072	11/02/2011	Healthy and colon cancer tissue	EVs from plants loaded with curcumin	Oral	Observational study	I	Recruiting
NCT05520125	29/08/2022	Bone tissue defects	MSCs enriched with EVs	Not stated	Standard treatment vs. Standard treatment + MSCs&EVs	I-II	Not yet recruiting
NCT05499156	12/08/2022	Resistant perianal fistula in Crohn’s disease	EVs from placental MSCs	Injection in surrounding tissue	Placebo vs. EVs	I-II	Active, not yet recruiting
NCT05402748	02/06/2022	Complex anal fistula	EVs from placental MSCs	Injection in the fistula tract	Placebo vs. EVs	I-II	Recruiting
NCT05127122	19/11/2021	ARDS	EVs from BMSCs (ExoFlo)	Intravenously	Placebo vs. 2 different doses of EVs	I-II	Not yet recruiting
NCT05116761	11/11/2021	Post-acute COVID-19 and Chronic post-COVID 19 syndrome	EVs from BMSCs (ExoFlo)	Intravenously	Placebo vs. EVs	I-II	Not yet recruiting
NCT04798716	15/03/2021	ARDS and COVID-19 pneumonia	EVs from perinatal MSCs	Intravenously	Placebo vs. escalating doses of EVs	I-II	Not yet recruiting
NCT04602104	26/10/2020	ARDS	EVs from MSCs	Inhaled	Placebo vs. 5 doses of EVs	I-II	Unknown
NCT04544215	10/09/2020	Resistant gram-negative bacilli pulmonary infection	EVs from ADSCs	Inhaled	Standard treatment vs. standard treatment + 2 doses of EVs	I-II	Recruiting
NCT04213248	30/12/2019	Dry eye in patients with graft versus host disease	EVs from umbilical cord-derived MSCs	Artificial tears	Single group pre-post study	I-II	Recruiting
NCT04173650	22/11/2019	Dystrophic epidermolysis bullosa	EVs from BMSCs (AGLE-102)	Topical	2 ascending dose levels of EVs	I-II	Not yet recruiting
NCT03384433	27/12/2017	Ischemic stroke	EVs from MSCs loaded with miR-124	Intraparenchymal	Placebo vs. EVs	I-II	Recruiting
NCT03230708	26/07/2017	Malignant ascites	EVs from erythrocytes loaded with methotrexate	Intraperitoneal	Standard treatment vs. standard treatment + EVs	I-II	Unknown
NCT02138331	14/05/2014	Type I diabetes	EVs from umbilical cord-derived MSCs	Intravenously	Control vs. EVs	I-II	Unknown
NCT05261360	02/03/2022	Degenerative Meniscal Injury	EVs from synovial fluid-derived MSCs	Intra-articular	Conservative treatment vs. EVs	II	Recruiting
NCT05125562	18/11/2021	Mild-to-moderate COVID-19	EVs from BMSCs (ExoFlo)	Intravenously	Placebo vs. 2 different doses of EVs	II	Not yet recruiting
NCT04969172	20/07/2021	Moderate-to-severe COVID-19	EVs over-expressing CD24 from T-REx-293 cells	Inhaled	Placebo vs. EVs	II	Active, not recruiting
NCT04902183	26/05/2021	Moderate-to-severe COVID-19	EVs over-expressing CD24 (CovenD24)	Inhaled	2 doses of EVs	II	Recruiting
NCT04602442	26/10/2020	COVID-19 pneumonia	EVs from MSCs	Inhaled	Placebo vs. 2 doses of EVs	II	Unknown
NCT04493242	30/07/2020	COVID-19 associated moderate-to-severe ARDS	EVs from BMSCs (ExoFlo)	Intravenously	Placebo vs. 2 different doses of EVs.	II	Completed, results not published. A 60-day mortality rate reduction of 37.6% was announced by the company
NCT05413148	09/06/2022	Retinitis pigmentosa	EVs from umbilical cord-derived MSCs	Sub-Tenon injection	Placebo vs. MSCs vs. EVs	II-III	Recruiting
NCT05216562	31/01/2022	Moderate COVID-19	EVs from MSCs	Intravenously	Placebo vs. EVs	II-III	Recruiting
NCT04761562	21/02/2021	Chronic Middle Ear Infections	Plasma rich in platelets and EVs	Locally	Tympanoplasty vs. tympanoplasty + EVs enriched plasma	II-III	Recruiting
NCT05354141	29/04/2022	Moderate-to-severe COVID-19-associated ARDS	EVs from BMSCs (ExoFlo)	Intravenously	Placebo vs. EVs	III	Recruiting
NCT05490173	05/08/2022	Neuroprotection in extremely low birth weight infants	EVs from MSCs	Intranasal	Placebo vs. EVs	N/A	Not yet recruiting
NCT05475418	26/07/2022	Wound healing	EVs from adipose tissue	Topical	Single group	N/A	Not yet recruiting
NCT04879810	10/05/2021	Inflammatory bowel disease	EVs derived from the ginger plant	Oral	Curcumin vs. EVs vs. EVs + curcumin	N/A	Completed
NCT04850469	20/04/2021	Intensively ill children	EVs from MSCs	Intravenously	Standard treatment vs. standard treatment + EVs	N/A	Withdrawn, lack of funding
NCT04657458	08/12/2020	COVID-19 associated ARDS	EVs from BMSCs (ExoFlo)	Intravenously	Expanded access protocol for NCT04493242	N/A	Available
NCT04652531	03/12/2020	Venous trophic lesions	Autologous serum-derived EVs	Topical	Standard treatment vs. standard treatment + EVs	N/A	Recruiting
NCT04356300	22/04/2020	Multiple organ dysfunction syndrome after surgical repair of acute type A aortic dissection	EVs from umbilical cord-derived MSCs	Intravenously	Standard treatment vs. standard treatment + EVs	N/A	Not yet recruiting
NCT04281901	24/02/2020	Chronic postsurgical temporal bone inflammations	Plasma rich in platelets and EVs	Locally	Conservative treatment vs. plasma	N/A	Completed, results published in [158]
NCT04223622	10/01/2020	Ex vivo osteoarthritis in osteochondral explants	EVs from ADSCs	Ex vivo	Observational study	N/A	Recruiting
NCT0402770	18/12/2019	Depression, anxiety, and dementia	EVs from cesarean section amniotic fluid	Intravenously	Single group	N/A	Suspended due to the COVID-19 pandemic
NCT03493984	11/04/2018	Polycystic ovary syndrome	EVs from ginger and aloe plants	N/A	N/A	N/A	Withdrawn
NCT02594345	03/11/2015	Ex vivo study on blood coagulation	EVs from red blood cells	Ex vivo	Observational study	N/A	Completed
